# Visible tunable lighting system based on polymer composites embedding ZnO and metallic clusters: from colloids to thin films

**DOI:** 10.1080/14686996.2016.1202724

**Published:** 2016-08-17

**Authors:** Thai Giang Truong, Benjamin Dierre, Fabien Grasset, Noriko Saito, Norio Saito, Thi Kim Ngan Nguyen, Kohsei Takahashi, Tetsuo Uchikoshi, Marian Amela-Cortes, Yann Molard, Stéphane Cordier, Naoki Ohashi

**Affiliations:** ^a^ Institut des Sciences Chimiques de Rennes (ISCR), UMR 6226, CNRS-University of Rennes 1, Rennes, France; ^b^ Laboratory for Innovative Key Materials and Structures (LINK), UMI 3629 CNRS-Saint Gobain-NIMS, National Institute for Materials Science (NIMS), Tsukuba, Japan; ^c^ Optical and Electronic Materials Unit, NIMS, Tsukuba, Japan; ^d^ NIMS-Saint-Gobain Center of Excellence for Advanced Materials, NIMS, Tsukuba, Japan; ^e^ Department of Metallurgy and Ceramics Science, Tokyo Institute of Technology, Tokyo, Japan; ^f^ Fine Particles Engineering Group, NIMS, Tsukuba, Japan; ^g^ Sialon Unit, NIMS, Tsukuba, Japan

**Keywords:** ZnO, metal clusters, luminescence, colloidal solution, thin films, 20 Organic and soft materials (colloids, liquid crystals, gel, polymers), 204 Optics / Optical applications, 301 Chemical syntheses / processing, 505 Optical / Molecular spectroscopy, 102 Porous / Nanoporous / Nanostructured materials, 103 Composites, 105 Low-Dimension (1D/2D) materials

## Abstract

The development of phosphor devices free of heavy metal or rare earth elements is an important issue for environmental reasons and energy efficiency. Different mixtures of ZnO nanocrystals with Cs_2_Mo_6_I_8_(OOC_2_F_5_)_6_ cluster compound (CMIF) dispersed into polyvinylpyrrolidone matrix have been prepared by very simple and low cost solution chemistry. The resulting solutions have been used to fabricate highly transparent and luminescent films by dip coating free of heavy metal or rare earth elements. The luminescence properties of solution and dip-coated films were investigated. The luminescence of such a system is strongly dependent on the ratios between ZnO and CMIF amounts, the excitation wavelength and the nature of the system. By varying these two parameters (ratio and wavelength), a large variety of colors, from blue to red as well as white, can be achieved. In addition, differences in the luminescence properties have been observed between solutions and thin films as well as changes of CMIF emission band maximum wavelength. This may suggest some possible interactions between the different luminophore centers, such as energy transfer or ligands exchange on the Mo_6_ clusters.

## Graphical Abstract

**Figure:**
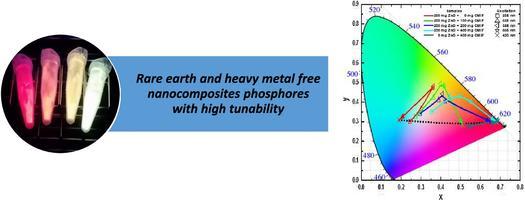

## 1. Introduction

More and more attention is being paid to environmental and energy efficiency issues,[1] e.g. the production of low carbon energy from ‘green’ resources, reducing energy consumption by improving device performances, or developing new more environmentally friendly materials. One of the largest potentials for energy savings is in the building sector, especially in energy efficient and innovative lighting concepts such as smart windows or light management. The US Department of Energy: http://energy.gov/sites/prod/files/2015/09/f26/QTR2015-05-Buildings.pdf reported that lighting accounts for approximately 18% of electricity use in US buildings, behind only space heating and cooling, and it could be reduced by half by improving lighting devices. In addition, the gradual phasing out of incandescent light bulbs ensures a more understanding and receptive market. A possible approach for these applications is to develop new phosphors, based on nanocomposite or hybrid materials, with improved properties (e.g. easy and low-cost synthesis, high transparency and efficiency, and less damaging to the environment) and high flexibility in their emission properties. New nanocomposite solutions or thin films may have potential applications, as well as in light devices [2–4] or luminescent solar concentrator for photovoltaic devices.[5] Chalcogenides quantum dots [6,7] and rare-earth (RE) doped materials [8–10] are considered as the most promising inorganic phosphor candidates. However, they may have some issues related to their toxicity in the case of quantum dots, which contain heavy metal (HM), or their high cost and environmental and availability problems due to their extraction in the case of RE doped materials. Moreover, most of these materials are limited to single narrow emission properties. Thus, other solutions, such as free HM and RE luminescent nanocrystal or metal clusters, may be also considered, as recently reported.[11–17]

It has been shown in a previous work that the association of zinc oxide (ZnO) nanocrystals and Cs_2_Mo_6_Br_14_ metal cluster compounds leads to colloidal materials with unique tunable emission properties.[18] Moreover, these two metals are not included in the list of European critical raw materials. Depending on the excitation wavelength, the resulting cluster@ZnO hybrid colloid showed either the visible emission of ZnO or the clusters emission, or even a combination of both, resulting in a broad emission covering almost the entire visible light range. More recently, ZnO-Cs_2_Mo_6_Br_14_ colloidal solutions were stabilized by addition of polyvinylpyrrolidone (PVP) in the presence of water and could be therefore used for the preparation of transparent and luminescent polymer thin films.[19] These films were transparent in the visible range, and produced a large emission band covering almost the entire visible range under excitation at 325 nm. Nevertheless, because of the low emission efficiency of the bromide cluster, the fine tuning of their luminescence properties was not possible. On the other hand, the increasing complexity of applications in the fields of light-emitting devices requires nanomaterials with more sophisticated emission properties. In particular, the achievement of materials with tunable and broad emission properties is of major interest for the manipulation of light over a large spectral range in the field of photonics.

Therefore, in this work, we used Cs_2_Mo_6_I_8_(C_2_F_5_COO)_6_ cluster compound (CMIF) in combination with ZnO nanocrystal luminophores embedded in PVP matrix as a visible tunable lighting system. Cs_2_Mo_6_I_8_(C_2_F_5_COO)_6_ cluster compound is based on [Mo_6_I_8_(C_2_F_5_COO)_6_]^2-^ molybdenum cluster units showing a higher quantum yield compared to values observed for their halide counterparts.[20–23] In this work, we investigated the luminescence properties of a mixture of ZnO nanocrystals and Cs_2_Mo_6_I_8_(OOC_2_F_5_)_6_ cluster-based compound dispersed into a PVP matrix in solution and dip-coated thin films. By varying the ratios between ZnO and CMIF, and the excitation wavelength, a large variety of emitted colors from blue to red, as well as white, was achieved. In addition, we also observed different emission behaviors of the mixtures in solution or in thin films, which suggests some interactions between the different luminescent centers.

## 2. Experimental procedures

The CMIF cluster compound was prepared from Cs_2_Mo_6_I_14_ and AgOCOC_2_F_5_. The compound integrity and purity were confirmed by ^19^F nuclear magnetic resonance measurements with the presence of only two signals at δ = –83 ppm and δ = –120 ppm, by energy-dispersive X-ray spectroscopy (EDX) and X-ray diffraction on single crystals. All data were consistent with previously published results.[20] The ZnO colloid was synthesized by a sol-gel route as described in a previously reported procedure.[24] The ZnO concentration of the colloidal solution in ethanol after purification was 0.5 M, as determined by inductively coupled plasma optical emission spectrometry (ICP-OES) measurements. The colloidal solutions of CMIF clusters and ZnO nanocrystals were prepared by dissolving an appropriate amount of CMIF (from 0 to 400 mg) in a mixture of acetone/ethanol. Then, 4 g of PVP (M_w_ = 40000 g mol^−1^) was dissolved in the resulting cluster solution. Finally, 5 ml of the ZnO colloidal solution was added to the mixture. The total volume was fixed at 20 ml for all the samples by adding 5 ml of acetone and the appropriate amount of ethanol. Table 1 shows the different compositions of samples. In the following, the samples will be designated by *x/y* referring to *x* mg and *y* mg amounts of ZnO and CMIF, respectively. It should be pointed out that PVP has been chosen as matrix because previous studies have already demonstrated that PVP can be efficiently used to synthesize or stabilize ZnO nanocrystals.[19,25–27] Due to its very low toxicity, PVP has been widely used for a long time as an excipient in pharmaceutical, foods or cosmetic applications.[28] More recently, it was used as polymer electrolyte for transparent thin film application,[29] coating on ZnO for the preparation of gas sensors,[30] or as an interlayer on a ZnO film for interfacial modification in inverted polymer solar cells.[31] A 15% enhancement in power conversion efficiency is realized after the incorporation of a PVP layer between ZnO and the photoactive layer in inverted polymer solar cells.

**Table 1.** Name, weights of ZnO, CMIF and PVP, and weight percentage of ZnO+CMIF of the different solutions.

Name	ZnO (mg)	CMIF (mg)	PVP (g)	Weight percentage of ZnO+CMIF (%)
0/0	0	0	4	0
200/0	200	0	4	4.76
200/100	200	100	4	9.97
200/200	200	200	4	9.10
200/400	200	400	4	13.04
0/400	0	400	4	9.10

From the stable colloidal solution, films were deposited on soda lime glass slides by dip-coating at room temperature. The solutions were placed in a Teflon® container and the substrate was introduced in the solution. After 1 min of immersion, the substrate was pulled up at rates ranging from 30 to 120 mm min^−1^. In the following, only the results for the 120 mm min^−1^ rate will be presented, as comparable results have been observed with the other pulling rates. After deposition, the films were dried at room temperature. The weight percentages of inorganic parts range from 0 to 13% (Table 1). Ultraviolet-visible spectroscopy (UV-vis) was performed in a spectrophotometer (V-570, Jasco, Tokyo, Japan) field emission, with solutions taken in absorbance mode with the same dilution and with films taken in transmission mode. Fluorescence properties were measured using a spectrofluorometer (FP8500, Jasco) with a 150 W Xe lamp with shielded lamp house as excitation source. The cross-sections of the films were observed using a field emission scanning electron microscope (FE-SEM, SU8000, Hitachi Ltd., Tokyo, Japan) at 10 kV. The luminescence spectra for quantum efficiency (QE) measurements were recorded using an intensified multichannel spectrometer (MCPD-7000, Otsuka Electronics, Tokyo, Japan) under ambient atmosphere at room temperature. XRD patterns were recorded by grazing incidence X-ray diffraction (GIXRD), to limit the substrate contribution, using a Rigaku SmartLab apparatus (Rigaku, Tokyo, Japan) equipped with a D/TeX Ultra 250 detector (Rigaku, Tokyo, Japan) and Cu radiation in the θ–θ configuration. Data were collected in the 10–60° 2θ range with a step of 0.02 and a speed of 1° min^–1^.

## 3. Results and discussion

### 3.1. Study of the colloidal solution

**Figure 1.**
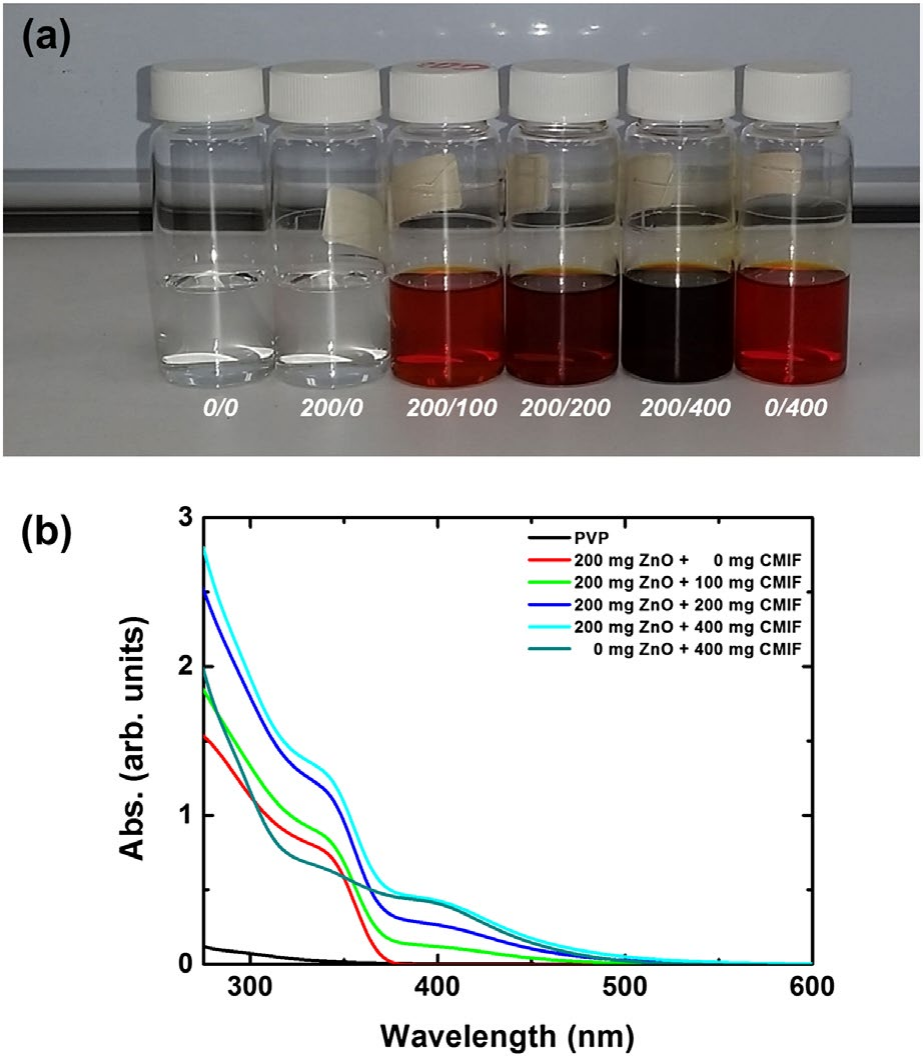
(a) Photograph of 0/0, 200/0, 200/100, 200/200, 200/400 and 0/400 solutions under visible light; (b) UV-vis spectra of 0/0, 200/0, 200/100, 200/200, 200/400 and 0/400 solutions.

Figure 1(a) shows a picture of 0/0, 200/0, 200/100, 200/200, 200/400 and 0/400 solutions under visible light. Clear solutions can be observed without haze or turbidity. This is related to the small size (<20 nm) and good dispersion of the ZnO nanocrystals and CMIF in solution, which reduce light scattering. It has been already shown that important optical scattering loss can be avoided if the size of the inorganic particles is less than one-tenth of the used light wavelength (300–800 nm). The 0/0 and 200/0 solutions are colorless, and the coloration of the solutions becomes redder by increasing the amount of CMIF, as expected. Figure 1(b) shows the UV-vis spectra of 0/0, 200/0, 200/100, 200/200, 200/400 and 0/400 solutions. By increasing the amount of CMIF, the absorption in the 250–550 nm region increases. More specifically, the absorption intensities at 400 nm are 0, 0.12, 0.26, 0.43 for the 200/0, 200/100, 200/200 and 200/400 solutions, respectively. Thus, the absorption at 400 nm is proportional to the quantity of CMIF in solution. Compared to the 200/400 solution, the 0/400 solution has a lower absorption in the UV region (from 250 to 375 nm) and a comparable one in the visible region. These results suggest that ZnO nanocrystals mainly absorb in the UV region (<360 nm due to quantum confinement effects), while CMIF absorbs in the UV and visible regions as expected. PVP has almost no absorption in the range 350–550 nm.

**Figure 2.**
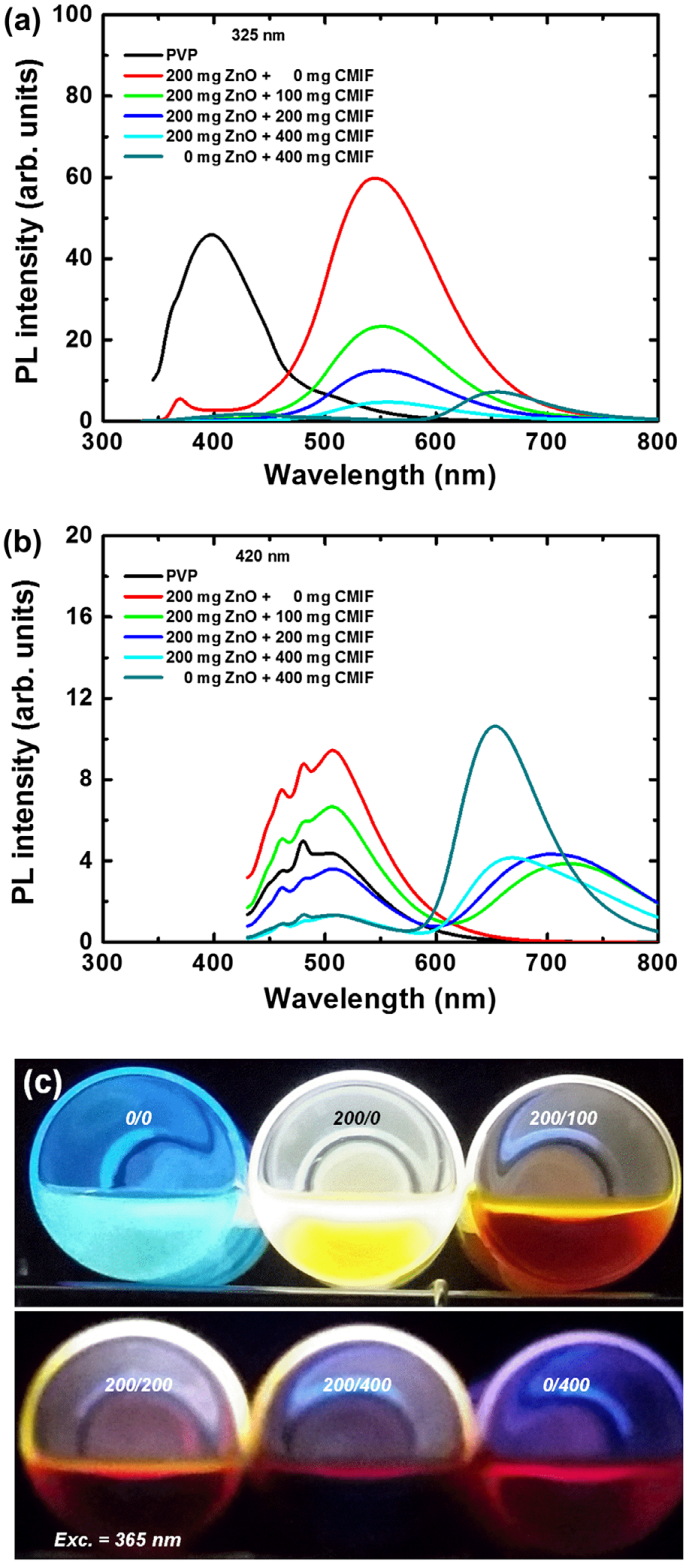
PL spectra for 0/0, 200/0, 200/100, 200/200, 200/400, 0/400 solutions under 325 (a) and 420 nm (b) excitations; (c) picture of the different solutions under 365 nm excitation.

Figure 2 shows the photoluminescence (PL) spectra for 0/0, 200/0, 200/100, 200/200, 200/400 and 0/400 solutions under 325 (a) and 420 nm (b) excitation, and a picture of the different solutions under 365 nm excitation (c). Under 325 nm excitation, the PL spectrum of PVP solution consists of a broad band at 400 nm with shoulders at 360 and 500 nm. With the incorporation of ZnO or CMIF, the PVP bands drastically decrease, while a strong band centered at 550 nm or 650 nm appears for 200/0 or 0/400 solutions, respectively. The 550 nm emission band is attributed to the photoluminescence generated by defects in ZnO nanocrystals,[32–37] while the 650 nm emission band is attributed to the [Mo_6_I^i^_8_(OOCC_2_F_5_)^a^_6_]^2-^ cluster units.[20] When the CMIF amount increases in the ZnO-containing solutions, no additional bands are observed, while the emission from ZnO nanocrystals decreases. The emission intensities at 550 nm are 60, 23, 12 and 4 for 200/0, 200/100, 200/200 and 200/400 solutions, respectively. Since the amount of ZnO has been kept constant, we can suppose that there may be interactions between the ZnO nanocrystals and the CMIF. Such interactions have already been observed in previous studies.[18,19] Indeed, the previously reported photoemission studies showed that [Mo_6_Br_14_]^2-^ cluster units can efficiently interact with ZnO nanocrystals, not only in a colloidal solution, but also in solid state conditions, confirming an immobilization of the units on the ZnO surface. Under 420 nm excitation, the PL spectrum of PVP solution consists of a broad band centered at 510 nm with sharp shoulders at 460 and 480 nm. By adding ZnO nanocrystals, no additional band appears as expected, although an increase of the PVP emission band is observed. With the incorporation of CMIF, a band in the red region appears, but surprisingly its maximum location is dependent on the amount of CMIF. These red bands are centered at 720, 710, 670 and 650 nm for 200/100, 200/200, 200/400 and 0/400 solutions, respectively. It is interesting to note that the intensities of the CMIF emission are comparable for 200/100, 200/200 and 200/400 solutions, while those of the PVP emissions decrease with the increase of CMIF amount. It suggests again some energy transfers and/or light reabsorption between PVP, ZnO and CMIF.

**Figure 3.**
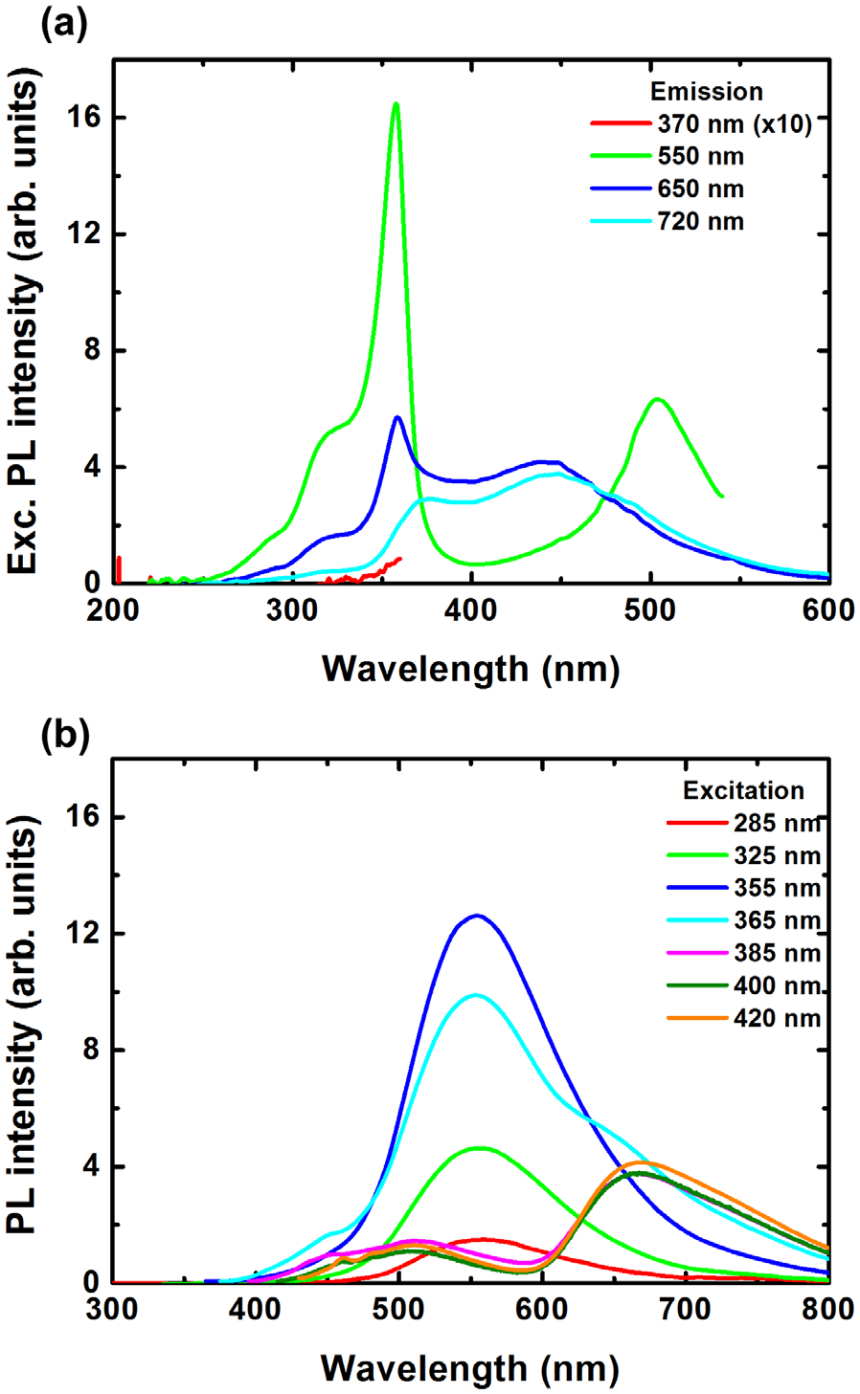
PL excitation (a) and emission spectra; (b) for 200/400 solution.

To investigate more in detail the luminescence properties of the solutions, we have studied the excitation spectra of the different emission bands and the emission spectra under different excitation wavelengths. Figure 3 shows the PL excitation (a) and emission spectra (b) for 200/400 solution. The excitation spectra were taken for 550, 650 and 720 nm emissions. For 550 nm emission, the PL excitation spectrum consists of relatively sharp bands at 320, 360 and 500 nm. For 650 nm emission, the PL excitation spectrum consists of weak bands at 320 and 360 nm, and broader bands at 380 and 450 nm. Finally, for 720 nm emission, the PL excitation spectrum only consists of the broader bands at 380 and 450 nm. By varying the excitation wavelength, the ratios of the different emission band intensities from PVP, ZnO and CMIF could be easily tuned. From 285 to 355 nm excitations, only the 550 nm bands related to ZnO emission is observed, and its intensity increases with the excitation wavelength. From 355 to 385 nm excitations, the 550 nm band intensity decreases and then disappears, while the intensities of bands centered at 450 and 510 nm, attributed to PVP, and at 670 nm, attributed to CMIF, increase. From 385 to 420 nm excitations, the PL spectra shapes are kept similar, while a slight decrease of the PVP bands and a slight increase of the CMIF bands are observed. Comparable tendency is observed for the other solutions.

**Figure 4.**
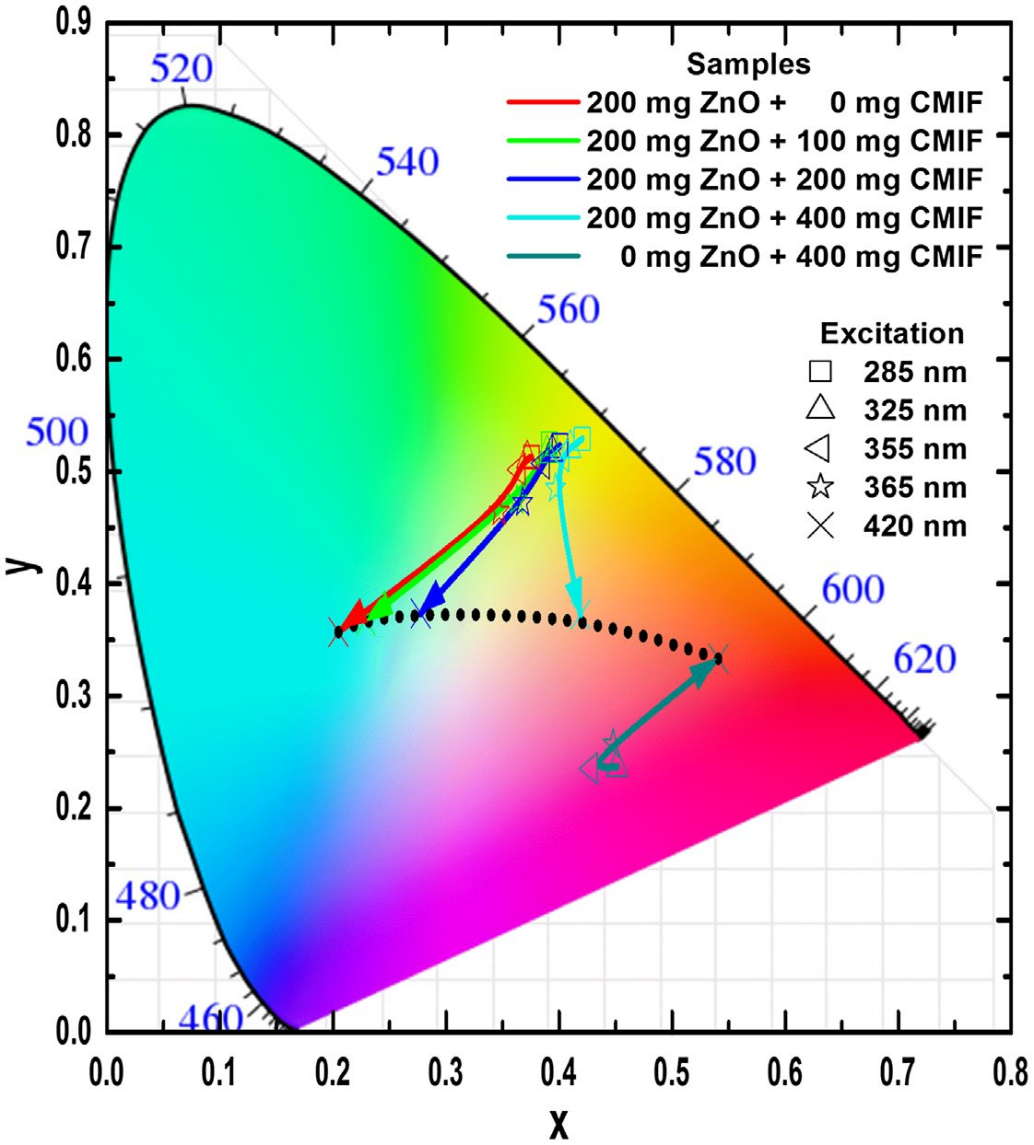
CIE chromaticity coordinates of 200/0, 200/100, 200/200, 200/400, 0/400 solutions under different excitation wavelengths.

In order to model the physiologically perceived colors in human color vision,[38] the International Commission on Illumination (CIE – Commission Internationale de l’Eclairage) has defined chromaticity coordinates. The latter have been determined for the studied colloidal solutions from the PL emission spectra of Figure 3. Figure 4 shows the CIE chromaticity coordinates of 200/0, 200/100, 200/200, 200/400 and 0/400 solutions under different excitation wavelengths. Under low wavelength excitation, the solutions containing ZnO and CMIF show relatively similar yellow-greenish color. By increasing the wavelength excitation, the color shifts to cyan for 200/0, to whitish for solutions containing small or medium amounts of CMIF, and yellow-orange for 200/400. For 0/400 solution, the color shifts from magenta to orange-red with the wavelength increase. It is very interesting to note that, under 420 nm excitation, it is possible to achieve cyan, white and orange-red lights by simply varying the ratio between ZnO and CMIF amounts, as shown by the dotted line.

### 3.2. Study of the PVP-based thin films

**Figure 5.**
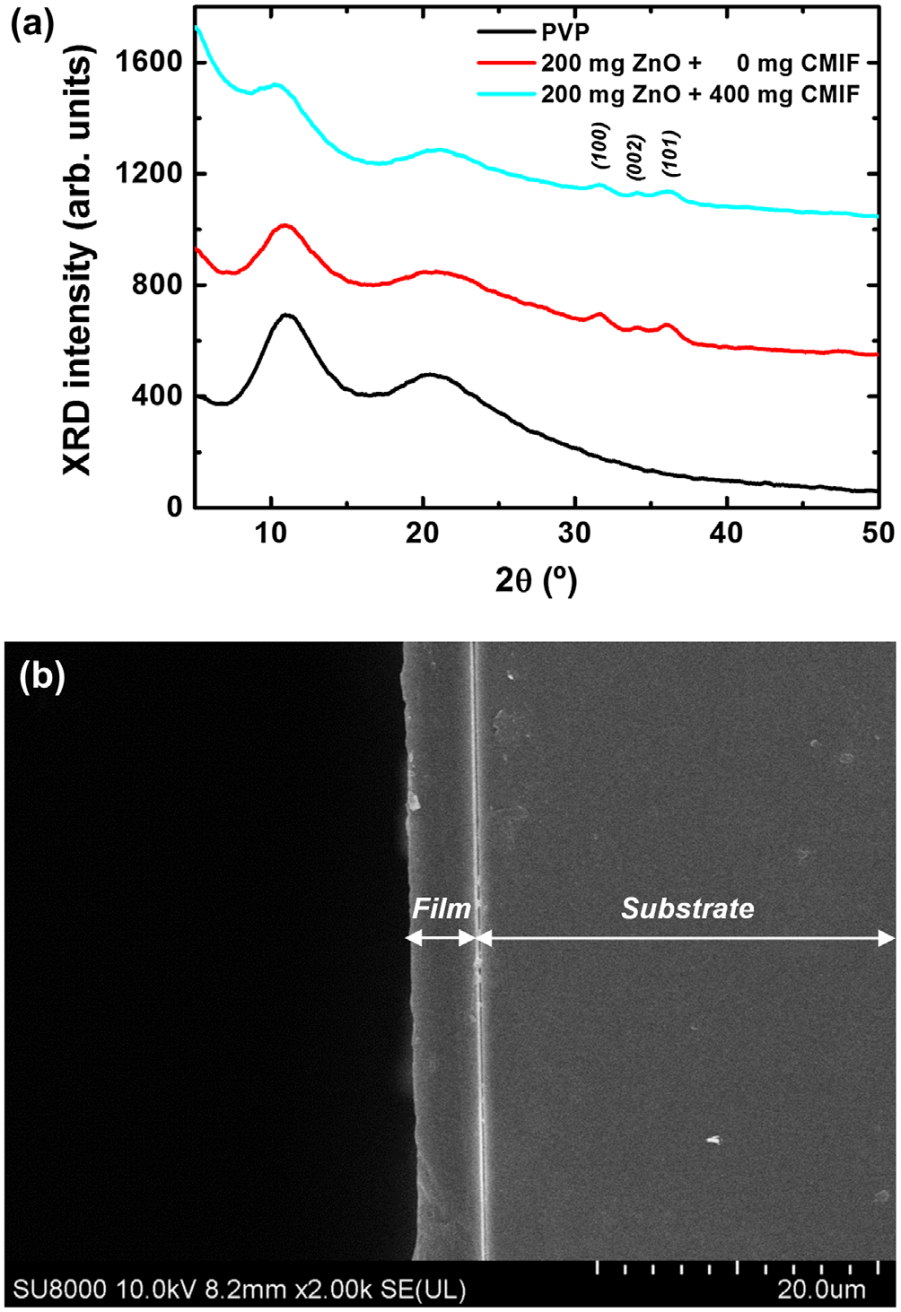
(a) XRD patterns for of 0/0, 200/0 and 200/400 films; (b) cross-sectional SEM image for 200/400 film.

The thin films prepared using solutions were first structurally analyzed to determine their uniformity. Figure 5(a) shows the XRD patterns of 0/0, 200/0 and 200/400 films. For the 0/0 film, the XRD pattern consists of two broad bands at 11.1 and 20.8°, attributed to the amorphous PVP. For the 200/0 film, in addition to the PVP bands, three small but clear peaks are observed at 31.8, 34.2 and 36.1°, which correspond to the (100), (002) and (101) peaks of wurtzite ZnO, respectively. The 200/400 film shows the same XRD pattern as the 200/0 film. In other words, no CMIF are observed by XRD due to the extremely small size of cluster compounds, close to 1 nm.[18] Figure 5(b) shows the cross-sectional SE image for the 200/400 film with a dipping speed of 120 mm min^−1^. The SEM image reveals a dense and quite homogeneous film with an average thickness of ~ 5 μm. Comparable results are obtained for the other films. It is possible to tune the thickness of the film by simply changing the dipping speed. The thickness of the film range from 1.8 to 5 μm when the dipping speed is increased from 30 to 120 mm min^−1^ speed rate (see Figure S1 in Supplementary Information).

**Figure 6.**
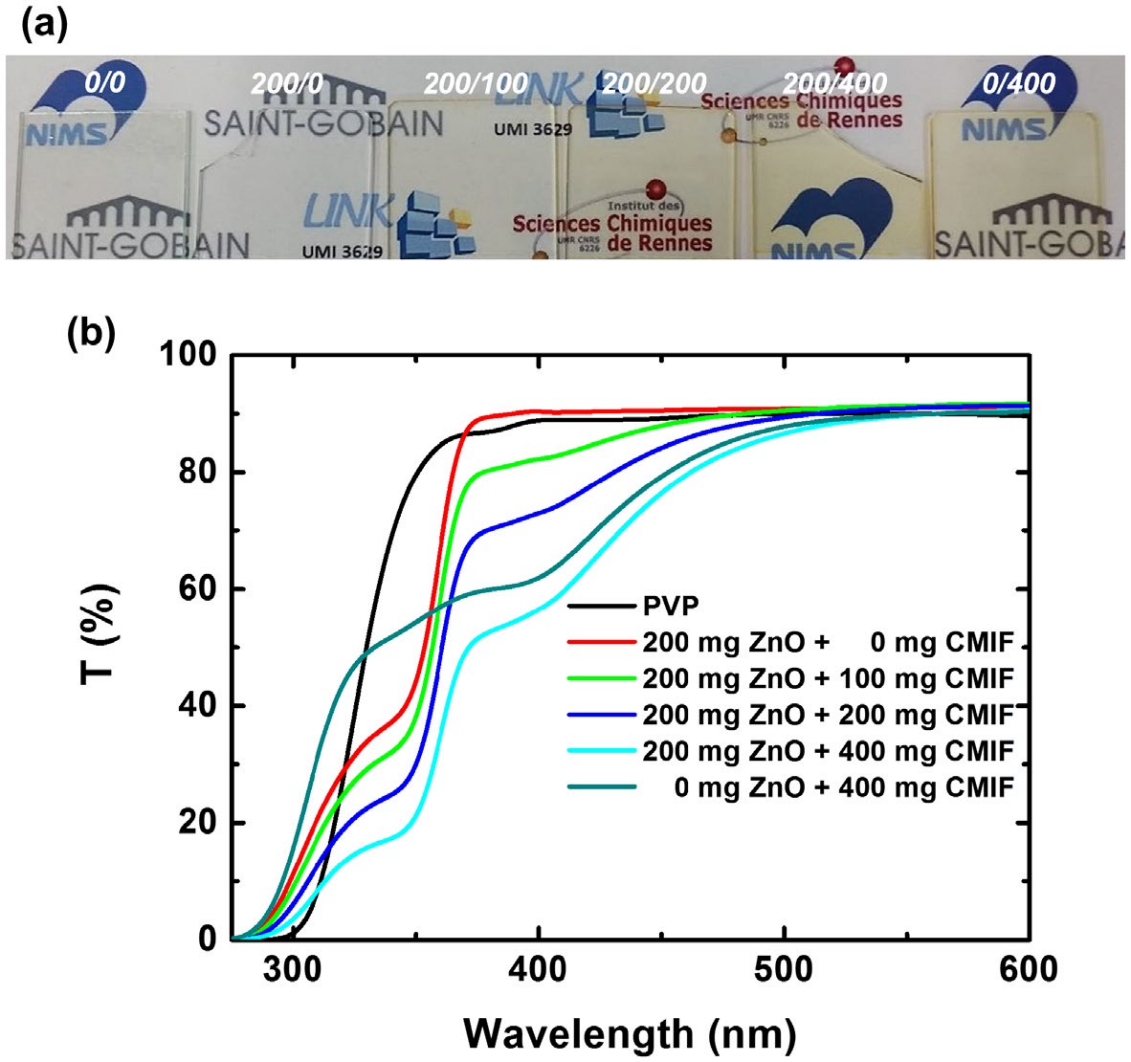
(a) Photograph of 0/0, 200/0, 200/100, 200/200, 200/400 and 0/400 films under visible light; (b) UV-vis spectra of 0/0, 200/0, 200/100, 200/200, 200/400 and 0/400 films.

Figure 6(a) shows a picture of 0/0, 200/0, 200/100, 200/200, 200/400 and 0/400 highly transparent films under visible light. The films containing only PVP or PVP+ZnO are colorless, and the film coloration becomes more orange-red by increasing the amount of CMIF. The excellent transmission (i.e. low scattering losses) for these nanocomposite thin film are again attributed to the good dispersion and small size of ZnO nanocrystals and nanosized [Mo_6_I^i^_8_(OOCC_2_F_5_)^a^_6_]^2-^ cluster units in the polymer matrix, as suggested by Rayleigh theory. Figure 6(b) shows the UV-vis spectra of 0/0, 200/0, 200/100, 200/200, 200/400 and 0/400 films. The 0/0 film absorbs under 300 nm and is 86% transparent from 370 nm. With the incorporation of ZnO, the optical band gap of the transmission film increases from 320 to 380 nm. It may be noted that while the 0/0 film starts to be transparent at 300 nm, the 200/0 is transparent from 290 nm. These results may be explained by a modification of the refractive index and absorption coefficient with the chemical composition. By increasing the amount of CMIF, the transmission decreases from 290 to 530 nm proportionally with the CMIF amount. Thus, the transmission intensities at 400 nm are 90, 82, 73 and 56% for the 200/0, 200/100, 200/200 and 200/400 solutions, respectively. Compared to the 200/400 film, the 0/400 solution has a lower absorption in the UV region (from 290 to 375 nm) and a comparable one in the visible region. The UV-vis results suggest that the ratios between ZnO and CMIF in the films are kept similar to that in the solutions.

**Figure 7.**
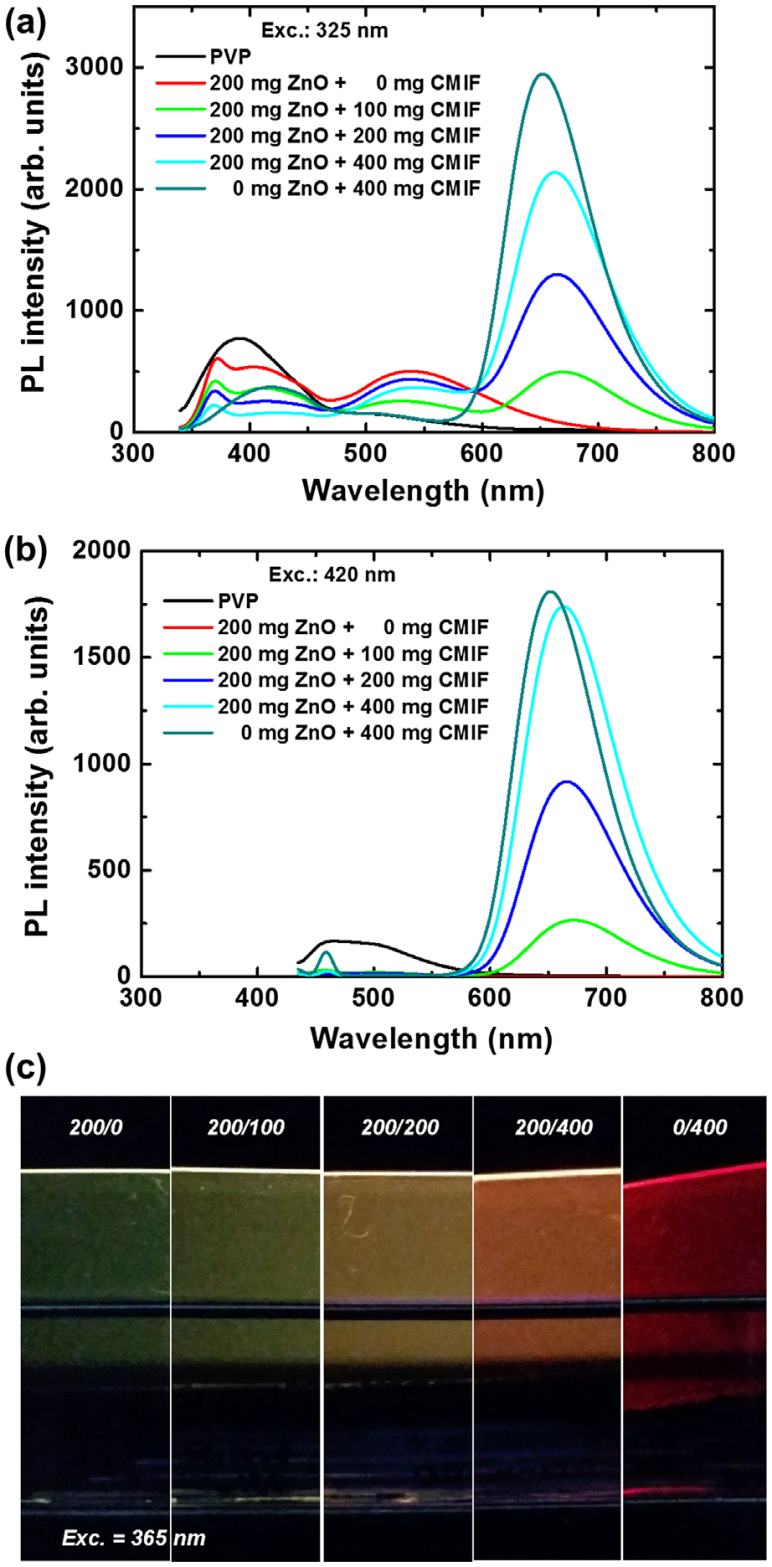
PL spectra for 0/0, 200/0, 200/100, 200/200, 200/400 and 0/400 films under 325 (a) and 420 nm (b) excitations; (c) photo of the different films under 365 nm excitation.

Figure 7 shows the PL spectra for 0/0, 200/0, 200/100, 200/200, 200/400 and 0/400 films under 325 (a) and 420 nm (b) excitations, and a picture of the different films under 365 nm excitation (c). Under 325 nm excitation, the PVP emission consists in a broad band at 390 nm and a smaller one at 510 nm. By incorporating ZnO, the band at 390 nm decreases, and a relatively sharp peak at 370 nm as well as bands at 420 and 550 nm are observed. The 370 nm emission is attributed to the near-band edge emission of ZnO, and the 420 nm one to PVP. When the amount of CMIF increases, the UV bands gradually decrease while the 550 nm band is kept relatively constant. A band around 670 nm gradually appears with the incorporation of CMIF. Interestingly, the position maximum of this band depends on the cluster concentration and moves from 673 nm to 663 nm by gradually increasing the cluster concentration within the mixture up to 653 nm for 0/400. For comparison, the solutions used to make these films have shown a strong emission at 550 nm and no CMIF emission except for 0/400. Under 420 nm excitation, the PVP film shows a broad band centered at 510 nm with a shoulder at 460 nm. With the incorporation of ZnO, these bands disappear. When CMIF is added, the main emission is centered on 670 nm. The red band under 420 nm excitation shows a similar cluster concentration dependency behavior to the one previously observed for an excitation at 325 nm. These results are different compared to those obtained in solution. A shift was also observed for the solutions, but from 720 to 650 nm. Moreover, in solutions, the main emission was observed at 550 nm, and was related to the defects of ZnO, and the CMIF emission was only observed under 420 nm excitation, while in the case of films, the main emission is the CMIF emission for both 325 and 420 nm excitations.

**Figure 8.**
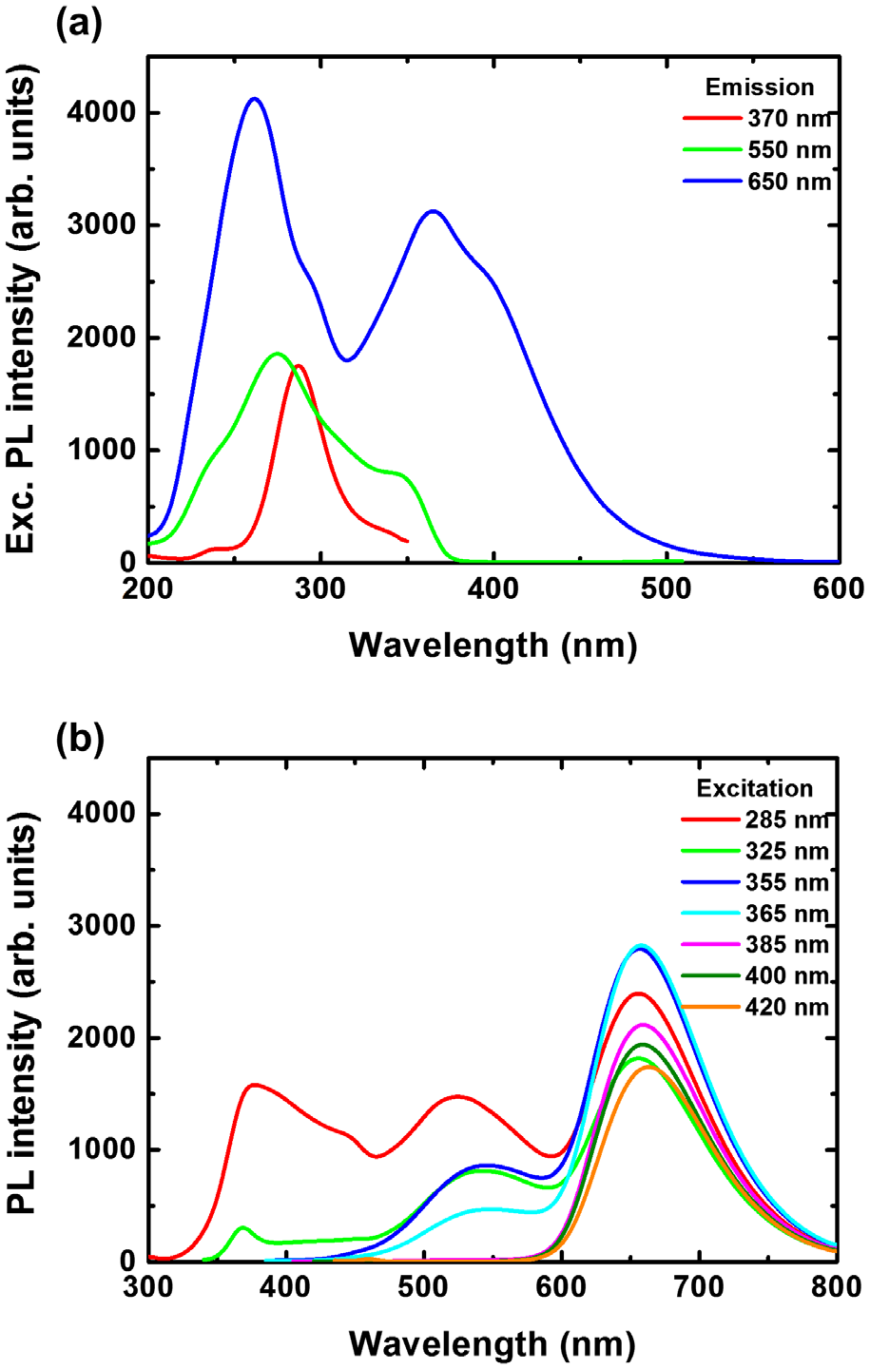
PL excitation (a) and emission spectra (b) for the 200/400 film.

We have investigated the excitation and emission properties of the different films under different emission and excitation wavelengths, respectively. Figure 8 shows the PL excitation (a) and emission spectra (b) for 200/400 film. The excitation spectra were taken for 550, 650 and 720 nm emissions. For 550 nm emission, the PL excitation spectrum consists of relatively sharp bands at 320, 360 and 500 nm. For 650 nm emission, the PL excitation spectrum consists of the weaker bands at 320 and 360, and broader bands at 380 and 450 nm. Finally, for 720 nm emission, the PL excitation spectrum only consists of the broader bands at 380 and 450 nm. By varying the excitation wavelength, the ratios of the different bands from PVP, ZnO and CMIF change. From 255 to 355 nm excitations, only the 550 nm band related to ZnO is observed, and its intensity increases with the excitation wavelength. From 355 to 385 nm excitations, the 550 nm bands decreases and then disappears, while the bands at 450 and 510, attributed to PVP, and at 670 nm, attributed to CMIF, increase. From 385 to 420 nm excitations, the spectral shapes of the PL spectra are constant, while a slight decrease of the PVP bands and a slight increase of the CMIF bands are observed. A comparable tendency is observed for the other films.

**Figure 9.**
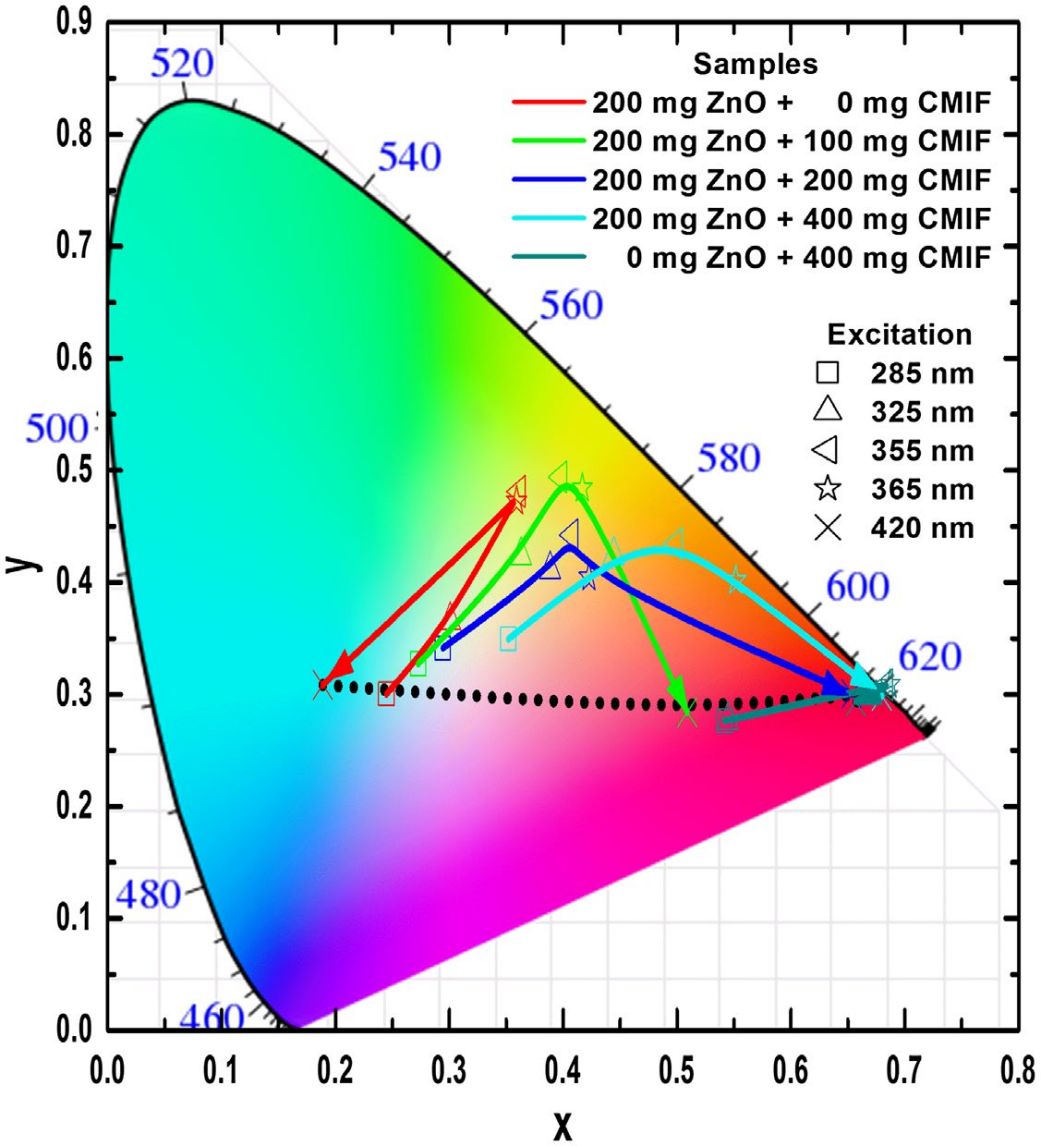
CIE chromaticity coordinates of 200/0, 200/100, 200/200, 200/400 and 0/400 films under different excitation wavelengths.

As for the solutions, the CIE coordinates have been determined from the PL emission spectra of Figure 8. Figure 9 shows the CIE chromaticity coordinates of 200/0, 200/100, 200/200, 200/400 and 0/400 films under different excitation wavelengths. Under low wavelength excitation, the solutions containing ZnO and CMIF show relatively similar yellow-greenish color. By increasing the wavelength excitation, the color shifts to cyan for 200/0, to whitish for films containing small or medium amounts of CMIF, and yellow-orange for 200/400. For 0/400 film, the color shifts from magenta to orange-red while the excitation wavelength increases. It is interesting to note that, under 420 nm excitation, it is possible to achieve cyan, white and orange-red colors by simply varying the ratios between ZnO and CMIF amounts, as shown in the dotted line.

**Figure 10.**
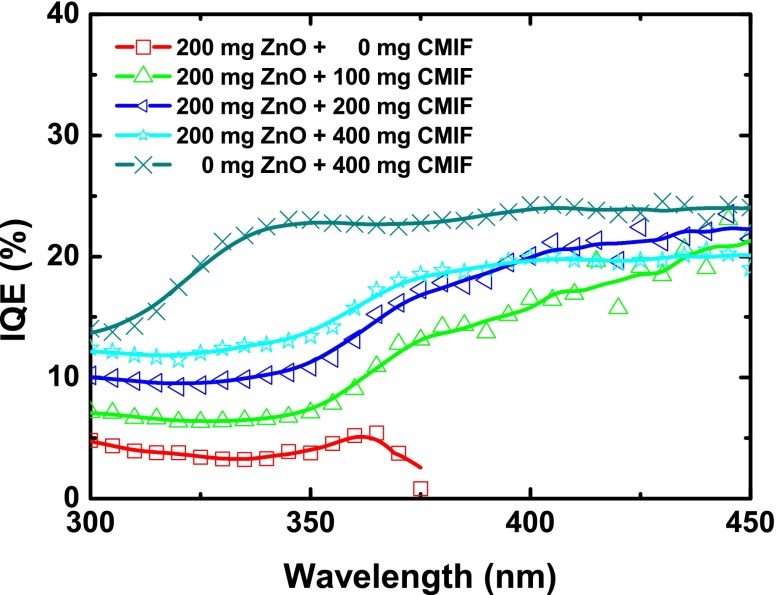
IQE of 200/0, 200/100, 200/200, 200/400 and 0/400 films under different excitation wavelengths.

Figure 10 shows the internal QE (IQE) of 200/0, 200/100, 200/200, 200/400 and 0/400 films under different excitation wavelengths. The wavelengths for at least 50% absorptance of 200/0, 200/100, 200/200, 200/400 and 0/400 films are 360, 365, 395, 440 and 425 nm, respectively. The IQE of 200/0 film is ~ 5% from 300 to 365 nm, and then decreases. For comparison, the IQE of ZnO nanocrystals in powder form (see Figure S2a in Supplementary Information) is around 14–18% from 300 to 400 nm. Such differences may be related to the small amount of ZnO into PVP and/or a modification of ZnO nanocrystals surface defects by the polymer or the chemical solutions. It may be supposed that a surface treatment of ZnO nanocrystals could prevent such effect. When CMIF is incorporated, the IQE increases. Interestingly, two regimes depending on the excitation wavelength are observed for all the samples containing both ZnO and CMIF: from 300 to 350 nm, the IQE is stable, around 7, 10 and 12% for 200/100, 200/200 and 200/400, respectively. For higher wavelength, the IQE increases to reach ~ 20% for all the samples. For the CMIF-only film (0/400), the IQE increases from 14% at 300 nm to 22% at 340 nm, and then is stable until 450 nm. For comparison, the IQE of CMIF powders measured in the same experimental conditions (Figure S2b in Supplementary Information) is ~ 19% from 300 to 400 nm and then decreases to 16% at 450 nm. Such IQE values for both powders and films are comparable to those of the previously reported nanocomposites.[20] The differences in the lower wavelength part may be related to the coexistence of different species, namely ZnO, PVP and soda lime glass. These results suggest that an improvement of the QE performance of CMIF is possible via dispersion into a matrix.

### 3.3. Comparison of emission properties of colloidal solutions and thin films

From these studies, we can assume that the luminescence of the ZnO+CMIF system in PVP is strongly dependent on the ratios between ZnO and CMIF amounts, the excitation wavelength and the nature of the system, namely solution or films. Indeed, as a function of these parameters, some luminescence centers are more excited compared to others. Thus, the green emission of ZnO nanocrystals is the dominant emission for colloidal solutions excited at 325 nm, while it is the red emission of CMIF which is dominant for films excited at 325 nm. Similarly, the emission of CMIF in solutions is not observed under excitation at 325 nm, but under excitation at 420 nm. These results suggest that some energy transfers between the different materials and/or reabsorption of the emitted light from PVP or ZnO by CMIF occur, in parallel with quenching by O_2_ dissolved in solution. In addition, the CMIF emission seems to be the most influenced by the ratios of the two phosphors. While for the emissions from PVP or ZnO, only the luminescence intensity is influenced by varying the amount of ZnO and CMIF, for the CMIF red emission, both intensities and peak positions are changed. Moreover, the peak shift is larger for the solutions (720 to 650 nm) than for the films (670 to 650 nm). This may be due to a partial replacement of the metal cluster pentafluoropropionate apical ligands by interactions with solvent or PVP. In addition, it may be possible that, depending on the amount of CMIF being incorporated, CMIF may occupy different locations in the system, such as being adsorbed on the ZnO surface, which may affect the distance between ZnO and CMIF, and consequently the interactions between them. Further experiments are in progress to explain this point.

## 4. Conclusions

Colloids of ZnO nanocrystals and Cs_2_Mo_6_I_8_(OOC_2_F_5_)_6_ (CMIF) cluster compound were prepared by very simple and low-cost solution chemistry and then dispersed into PVP. The resulting solutions have been used to fabricate highly transparent and luminescent films by dip coating free of heavy metals or rare earth elements. The luminescence of this system is strongly dependent on the ratios between ZnO and CMIF amounts, the excitation wavelength and the nature of the system. Thus, a large variety of emission colors from blue to red, and white, is achieved. In addition, differences in the luminescence properties have been observed between colloidal solutions and thin films as well as changes of CMIF emission band maximum position. This may suggest some possible interactions between the different luminophores, such as energy transfer or ligands exchange on the Mo_6_ clusters.

## Disclosure statement

No potential conflict of interest was reported by the authors.

## Funding

This work is funded by Center of Excellence NIMS-Saint Gobain and Institut de Chimie, CNRS.

## Supplemental material

The supplemental material for this paper is available online at http://dx.doi.org/10.1080/14686996.2016.1202724.

## Supplementary Material

Supporting_Information.docx
