# Supplementary material for: Visible tunable lighting system based on polymer composites embedding ZnO and metallic clusters: from colloids to thin films
Source: Sci Technol Adv Mater. 2016 Aug 17;17(1):443–53. doi: 10.1080/14686996.2016.1202724 (PMC5101959; doi:10.1080/14686996.2016.1202724)
Supplement: Supporting_Information.docx [file tsta_a_1202724_sm3613.docx]

**SUPPORTING INFORMATION**

**Visible tunable lighting system based on polymer composites embedding ZnO and metallic clusters: from colloids to thin films**

Thai Giang Truong^1,2,3^, Benjamin Dierre^2,4^*, Fabien Grasset^2,3,4^*, Noriko Saito^3^, Norio Saito^3,5^, Thi Kim Ngan Nguyen^6^, Kohsei Takahashi^7^, Tetsuo Uchikoshi^2,6^, Marian Amela-Cortes^1^, Yann Molard^1^, Stéphane Cordier^1^*, Naoki Ohashi^2,3,4^

# ^1^Institut des Sciences Chimiques de Rennes (ISCR), UMR 6226, CNRS-University of Rennes 1, Rennes, France

# ^2^Laboratory for Innovative Key Materials and Structures (LINK), UMI 3629 CNRS-Saint Gobain-NIMS, National Institute for Materials Science (NIMS), 1-1 Namiki. Tsukuba, Japan

# ^3^Optical and Electronic Materials Unit, NIMS, 1-1 Namiki, Tsukuba, Japan

^4^NIMS-Saint-Gobain Center of Excellence for Advanced Materials, NIMS, 1-1 Namiki, Tsukuba, Japan

^5^Department of Metallurgy and Ceramics Science, Tokyo Institute of Technology, 2-12-1 Ookayama, Tokyo, Japan

^6^Fine Particles Engineering Group, NIMS, 1-2-1 Sengen, Tsukuba, Japan

^7^Sialon Unit, NIMS, 1-1 Namiki, Tsukuba, Japan

*Corresponding authors : E-mail: [Dierre.Benjamin@nims.go.jp](mailto:Dierre.Benjamin@nims.go.jp) ; [Grasset.Fabien@nims.go.jp](mailto:Grasset.Fabien@nims.go.jp) ; Stephane.cordier@univ-rennes1.fr

**
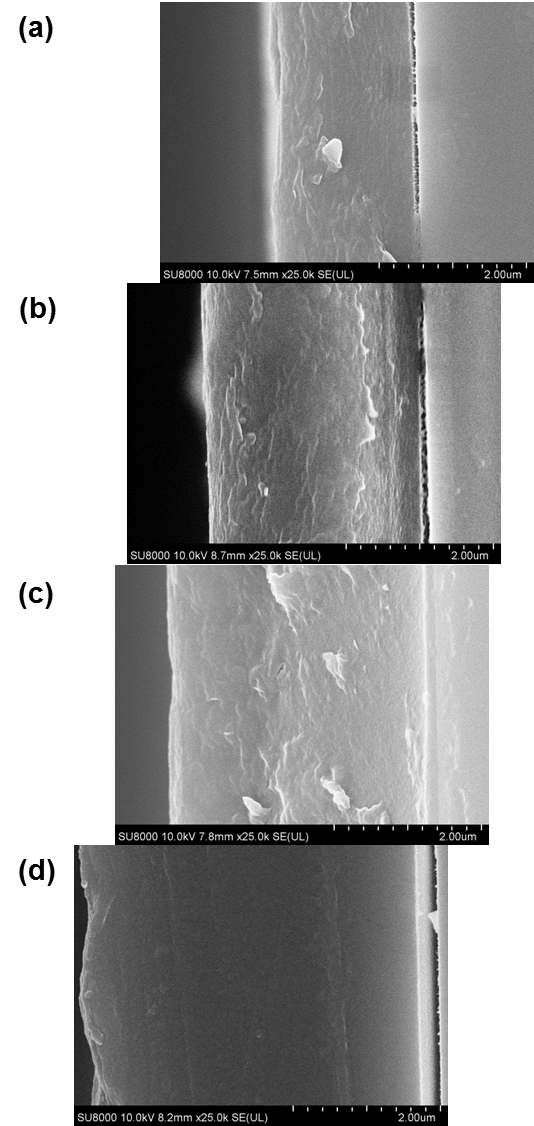
**

**Fig. S1.** Cross-sectional SE image for 200/400 film with a dipping speed rate of 30 (a), 60 (b), 90 (c) and 120 mm.min^-1^ (d).

**Fig. S2.** IQE of ZnO nanocrystals (a) and CMIF (b) in powder form under different excitation wavelengths.
